# Evaluating the effectiveness of the ‘eco-cooler’ for passive home cooling

**DOI:** 10.1038/s44168-024-00165-7

**Published:** 2024-11-02

**Authors:** Aditi Bunker, Karin Lundgren Kownacki, Sudipa Sarker, Rahmatul Bari, Malabika Sarker, Jonathan J. Buonocore, Pascal Geldsetzer, Johan Revstedt, Till Bärnighausen

**Affiliations:** 1 Heidelberg Institute of Global Health (HIGH), Heidelberg University, Heidelberg, Germany; 2 Swedish Meteorological and Hydrological Institute, Norrköping, Sweden; 3 The Business School, Edinburgh Napier University, Edinburgh, UK; 4 James P Grant School of Public Health, BRAC University, Dhaka, Bangladesh; 5 School of Public Health, Boston University, Boston, MA USA; 6 Division of Primary Care and Population Health, Department of Medicine, Stanford University, Stanford, CA USA; 7 Department of Energy Sciences, Lund University, Lund, Sweden; 8 Africa Health Research Institute (AHRI), KwaZulu-Natal, South Africa; 9 Department of Global Health and Population, Harvard T.H. Chan School of Public Health, Boston, MA USA

**Keywords:** Engineering, Environmental sciences, Developing world

## Abstract

Constructed with used plastic bottles, the eco-cooler has been widely adopted in resource-poor communities in Bangladesh and other countries. We tested the eco-cooler under controlled conditions using a scientific wind tunnel in a climatic chamber. In our tests, we used seven eco-cooler designs in 27 climate conditions typical of Bangladesh (temperatures of 40 °C, 35 °C, and 30 °C; humidity levels of 70%, 60%, and 40%; and wind speeds of 4.0 m s^−1^, 2.0 m s^−1^, and 0.2 m s^−1^) in 92 experiments (*N* = 7686 measurements in 87 short experiments and *N* = 23,428 measurements in five long experiments). We found no significant temperature reductions with eco-cooler use, except at low wind speeds, where temperature reduced by up to 0.2 °C. In theoretical calculations extending our empirical findings, the greatest temperature drop was 0.85 °C at 4.0 m s^−1^ with a 40 °C static air inflow temperature. However, this temperature drop did not extend beyond the nozzles of the bottles in the eco-cooler. The eco-cooler did not work effectively as an indoor air cooler.

## Introduction

Frugal innovation is often simple, leaving out features that are not necessary for a product to fulfill its essential function^1^. Because of their simplicity, frugal innovations can be resource- and cost-efficient in production, as well as affordable to people living in poverty or even extreme poverty^2–5^. Described as an effective passive home cooler suitable for resource-poor settings, the ‘eco-cooler’ is a frugal innovation that seems appealing because it is based on local knowledge^5^ and uses materials that are low-cost and readily available even in extremely resource-poor communities^6^. The eco-cooler was developed in a collaboration between a Social Business and an advertising agency^7^ to provide passive home cooling. The eco-cooler is constructed using discarded plastic bottles that are cut in half to create funnels^8^. The nozzle end of the bottle is embedded into a board that fits into a window frame flush with the wall. Gaining prominence through social and traditional media, advertising for the eco-cooler claimed that the device lowers indoor temperature by up to 5 °C by acting as a funnel to propel air forward and cool the air^8,9^. A total of 25,000 eco-coolers were reportedly installed across five rural Bangladeshi villages—Nilphamari, Daulatdia, Paturia, Modonhati and Khaleya^9^.

Since the release of the eco-cooler advertising campaign in 2016, the general product overview information has spread beyond Bangladesh, and basic news coverage can be found in English, Arabic, Spanish, Turkish and Vietnamese, among others^10,11^. The popularity of the eco-cooler indicates a general demand for the development and promotion of passive cooling devices for homes in resource-poor communities. The World Economic Forum, for example, publicized another passive cooling device, a zero-energy cooler that uses water evaporation to cool the surrounding air^12^. The use of water and the large size of the zero-energy cooler render this device impractical for home cooling. The social media attention for the zero-energy cooler (including a promotional video that has been shared 51,000 times and viewed 5.6 million times on Facebook since September 2017) demonstrates the strong public interest in locally developed, simple innovations for complex problems.

Across Bangladesh and other developing countries, the extensive adoption of heat-absorbing building materials, such as corrugated tin or iron, leads to high indoor temperatures in the summer months^13,14^. Because people in resource-poor communities may find it difficult to afford and access energy-consuming and expensive technologies for cooling, such as air conditioners, these housing conditions create a need for affordable and accessible passive cooling technologies —to ease human discomfort and reduce disease burdens caused by heat. This need will grow because ambient air temperature will likely increase with climate change in the coming decade^15^.

Prior to the use of the eco-cooler in homes, little scientific testing of the technology had been conducted, even though such testing should precede field tests and real-life use of novel products^16,17^. Of five studies that tested the technology after adoption in real life, each found lower changes in temperatures than what had been claimed in the media, with widely varying effects. Across the studies, average temperature reductions ranged from 0.2 °C to 4.0 °C^18–22^. Further, each of these earlier studies was conducted in ‘home-made’ laboratories where it is not possible to simultaneously and reliably control ambient temperature, humidity, and wind speed—such control, however, is necessary to reliably evaluate the performance of passive cooling devices.

We thus conducted tests of the eco-cooler under the controlled conditions of a scientific wind tunnel in a climatic chamber. We placed the eco-cooler into a wind tunnel to control air velocity; the surrounding climatic chamber allowed us to control ambient temperature and humidity. Our approach enabled us to simultaneously, reliably and precisely control all three climate conditions affecting the performance of the eco-cooler^23^—ambient temperature, humidity, and wind speed—thus overcoming the critical limitation of the previous tests^18–22^. We simulated Bangladeshi weather conditions (Supplementary Table 1)—which are similar to those in many other hot, wet, and humid tropical world regions—and systematically varied ambient temperature, humidity, and wind speed. We further tested the eco-cooler with different bottle designs^24^. We complemented our controlled experiments with two additional studies: an open-environment laboratory study, as an additional robustness check, and a theoretical model, to extrapolate our findings to climate conditions that were too extreme to be created in the wind tunnel.

## Results

### Eco-cooler effects on temperature, humidity, wind speed and atmospheric pressure

We tested the eco-cooler under the controlled conditions of a scientific wind tunnel in a climatic chamber. The full experimental setup is detailed in the methods section. The eco-cooler did not substantially reduce indoor temperature under any of our wide range of ambient temperatures, humidity levels, and wind speeds (Table 1). In the climate chamber, eco-cooler use resulted in a maximum temperature increase of 0.20 °C under two medium wind speed conditions respectively (i) 2.0 m s^−1^, 30 °C, 70% humidity, and (ii) 2.0 m s^−1^, 40 °C, 60% humidity. Eco-cooler use cooled temperature up to 0.20 °C in chamber conditions of 40 °C and 40% humidity—when the wind speed was low (0.2 m s^−1^). In seven out of nine tests, small temperature reductions of up to 0.2 °C were registered only at this low wind speed.

**Table 1:** Eco-cooler effects on temperature, humidity, wind speed and atmospheric pressure

	Eco-cooler effect estimates				
Experiment number and chamber condition	Number of measurements (*N*)	Temperature ( °C)	Humidity (%)	Wind speed (m/s)	Atmospheric pressure (bar)
40 °C, 70% humidity					
4.0 m/s	126	0.00 (−0.21–0.21) 0.984	−0.40 (−1.50–0.70) 0.470	2.59	0.002
2.0 m/s	126	0.01 (−0.10–0.13) 0.855	−0.38 (−1.88–1.12) 0.614	1.31	0.000
0.2 m/s	126	−0.18 (−0.20 to −0.16) <0.001	0.33 (−0.53–1.19) 0.443	0.20	0.000
35 °C, 70% humidity					
4.0 m/s	126	0.14 (−0.02–0.30) 0.095	−0.85 (−1.16 to −0.54) <0.001	2.55	0.002
2.0 m/s	126	0.17 (0.10–0.24) <0.001	−0.87 (−1.08 to −0.67) <0.001	1.13	0.000
0.2 m/s	126	−0.06 (−0.08–0.04) <0.001	−0.13 (−0.78–0.51) 0.678	0.20	0.000
30 °C, 70% humidity					
4.0 m/s	126	0.13 (−0.10–0.36) 0.253	−0.84 (−1.51 to −0.17) 0.0154	2.67	0.002
2.0 m/s	126	0.20 (0.15–0.26) <0.001	−1.01 (−1.34 to −0.68) <0.001	1.10	0.000
0.2 m/s	126	−0.03 (−0.04 to −0.02) <0.001	−0.21 (−0.87–0.44) 0.512	0.20	0.000
40 °C, 60% humidity					
4.0 m/s	126	0.04 (−0.08–0.14) 0.585	−0.33 (−1.79–1.14) 0.656	2.82	0.002
2.0 m/s	126	0.20 (0.15–0.26) <0.001	−1.01 (−1.34 to −0.68) <0.001	1.70	0.000
0.2 m/s	126	−0.08 (−0.12 to −0.05) <0.001	0.17 (−0.28–0.63) 0.440	0.20	0.000
35 °C, 60% humidity					
4.0 m/s	126	0.09 (−0.06–0.24) 0.226	−0.54 (−2.31–1.24) 0.543	2.98	0.002
2.0 m/s	126	0.04 (−0.04–0.12) 0.320	−0.35 (−1.39–0.70) 0.504	1.50	0.000
0.2 m/s	126	−0.02 (−0.09–0.05) 0.547	0.07 (−0.19–0.33) 0.575	0.20	0.000
30 °C, 60% humidity					
4.0 m/s	126	0.08 (−0.05–0.22) 0.229	−0.55 (−1.68–0.59) 0.339	2.88	0.002
2.0 m/s	126	0.11 (0.08–0.14) <0.001	−0.56 (−0.90 to −0.22) 0.002	1.36	0.000
0.2 m/s	126	−0.07 (−0.08 to −0.05) <0.001	0.07 (−0.58–0.73) 0.820	0.20	0.000
40 °C, 40% humidity					
4.0 m/s	126	−0.05 (−0.09–0.00) 0.062	−0.09 (−0.23–0.06) 0.218	3.11	0.002
2.0 m/s	126	0.00 (−0.03–0.03) 0.961	−0.13 (−0.52–0.27) 0.526	1.61	0.000
0.2 m/s	126	−0.20 (−0.25 to −0.16) <0.001	−0.01 (−1.95–1.93) 0.989	0.20	0.000
35 °C, 40% humidity					
4.0 m/s	126	0.05 (−0.03–0.13) 0.180	−0.32 (−0.81–0.18) 0.204	2.89	0.002
2.0 m/s	126	0.07 (0.06–0.08) <0.001	−0.31 (−0.42 to −0.20) <0.001	1.53	0.001
0.2 m/s	126	−0.08 (−0.10 to −0.07) <0.001	0.22 (0.13–0.32) <0.001	0.20	0.000
30 °C, 40% humidity					
4.0 m/s	126	0.06 (−0.02–0.14) 0.123	−0.35 (−0.44 to −0.30) <0.001	2.86	0.002
2.0 m/s	126	0.13 (0.10–0.17) <0.001	−0.45 (−0.87 to −0.03) 0.036	1.35	0.000
0.2 m/s	126	−0.07 (−0.1–0.0) <0.001	0.02 (−0.10–0.15) 0.731	0.20	0.000

The effects of the eco-cooler on temperature and humidity were measured with six sensors placed in the climate chambers. Four of these sensors were placed before the eco-cooler and four after the eco-cooler. We used linear regression to quantify the eco-cooler effect (i.e., the difference between the sensor measurements before and after the eco-cooler). The numbers shown for each eco-cooler design and climate scenario are (from right to left in each cell): the eco-cooler average effect size, and the 95% confidence interval (in parentheses) and the p value of the effect size.

Wind speed and atmospheric pressure estimates were empirical spot measurements made by using a probe.

High humidity = 70%, medium humidity = 60%, low humidity = 40%.

High temperature = 40 °C, medium temperature = 35 °C, low temperature = 30 °C.

Tests consisted of three wind speeds: 4 m/s, 2 m/s, and 0.2 m/s.

The error margin is ±0.5 °C for the temperature sensor and ±4.5% for and the humidity sensor.

Our experiments also revealed that the eco-cooler only minimally affected humidity. Eco-cooler use reduced humidity by 0.1% to 1.0% for most combinations of ambient temperatures, humidity levels, and wind speeds. Under the highest temperature and humidity settings (40 °C, 70%), the eco-cooler reduced humidity by 0.4%. Humidity reduced by 1.01% with eco-cooler use in two climate chamber conditions: (i) 2.0 m s^−1^ wind speed, 40 °C and 60% humidity and (ii) 2.0 m s^−1^ wind speed, 30 °C and 70% humidity. High or medium wind speeds (4.0 m s^−1^ and 2.0 m s^−1^) resulted in minute humidity reductions.

Humidity was very stable across different bottle designs (Table 2). The largest increase in humidity with eco-cooler use was 0.63% at 35 °C, 60% humidity with bottle designs 3 and 6. The greatest decrease in humidity was 0.67% at 4.0 m s^−1^ wind speed, 40 °C and 70% humidity.

**Table 2:** Eco-cooler effects on temperature, humidity, wind speed and atmospheric pressure for six eco-cooler designs

	Design 1: larger bottles		Design 2: smaller bottles		Design 3: flipped board (suction)		Design 4: nozzle extension		Design 5: reduced air resistance		Design 6: mixing air at nozzle	
Short tests												
Duration (minutes)	20		20		20		20		20		20	
Number of measurements per experiment (*N*)	126		126		126		126		126		126	
40 °C, 70% humidity	Eco-cooler effect estimates for temperature ( °C)											
4.0 m/s	0.00 (−0.21–0.21) 0.984		0.11 (0.04–0.18) 0.179		0.02 (−0.02–0.05) 0.357		0.11 (0.02–0.20) 0.016		0.20 (−0.43–0.83) 0.522		0.11 (0.06–0.16) <0.001	
2.0 m/s	0.01 (−0.10–0.13) 0.855		0.11 (0.00–0.21) 0.042		0.02 (−0.08–0.12) 0.662		0.13 (0.09–0.17) <0.001		0.10 (0.04–0.17) 0.002		0.11 (0.02–0.19) 0.012	
0.2 m/s	−0.18 (−0.20 to −0.16) <0.001		−0.04 (−0.04–0.11) 0.329		−0.05 (−0.06–0.04) <0.001		−0.17 (−0.22 to −0.11) <0.001		−0.08 (−0.11 to −0.06) <0.001		−0.13 (−0.20 to −0.15) <0.001	
40 °C, 70% humidity	Eco-cooler effect estimates for humidity (%)											
4.0 m/s	−0.40 (−1.50–0.70) 0.470		−0.47 (−1.45–0.51) 0.338		−0.00 (−0.99–0.98) 0.995		−0.42 (−0.85–0.01) 0.057		−0.67 (−1.76–0.42) 0.221		−0.40 (−0.90–0.13) 0.139	
2.0 m/s	−0.38 (−1.88–1.12) 0.614		−0.44 (−0.94–0.06) 0.086		−0.05 (−1.24–1.13) 0.928		−0.43 (−2.01–1.15) 0.585		−0.33 (−1.95–1.29) 0.683		−0.33 (−1.08–0.42) 0.382	
0.2 m/s	0.33 (−0.53–1.12) 0.614		−0.23 (−0.98–0.52) 0.533		0.17 (−0.73–1.07) 0.704		0.42 (−0.03–0.86) 0.066		0.22 (−1.15–1.58) 0.751		0.40 (−1.23–2.02) 0.622	
35 °C, 60% humidity	Eco-cooler effect estimates for temperature ( °C)											
4.0 m/s	0.09 (−0.06–0.24) 0.226		0.11 (−0.12–0.35) 0.334		−0.02 (−0.28–0.24) 0.870		0.13 (0.05–0.20) 0.001		0.08 (0.02–0.14) 0.009		0.09 (0.01–0.17) 0.031	
2.0 m/s	0.04 (−0.04–0.12) 0.320		0.12 (−0.01–0.24) 0.073		0.02 (−0.14–0.19) 0.775		0.11 (0.05–0.17) <0.001		0.05 (−0.10–0.19) 0.508		0.08 (−0.00–0.17) 0.625	
0.2 m/s	−0.02 (−0.09–0.05) 0.547		0.02 (−0.01–0.04) 0.269		−0.07 (−0.09 to −0.06) <0.001		−0.16 (−0.2 to −0.1) <0.001		−0.10 (−0.14 to −0.06) <0.001		−0.15 (−0.20 to −0.10) <0.001	
35 °C, 60% humidity	Eco-cooler effect estimates for humidity (%)											
4.0 m/s	−0.54 (−2.31–1.24) 0.543		−0.55 (−1.5–0.50) 0.295		0.09 (−1.65–1.84) 0.917		−0.48 (−1.07–0.11) 0.105		−0.31 (−2.86–2.25) 0.810		−0.32 (−0.57 to −0.08) 0.012	
2.0 m/s	−0.35 (−1.39–0.70) 0.504		−0.49 (−1.04–0.06) 0.079		−0.06 (−0.72–0.59) 0.846		−0.36 (−0.84–0.12) 0.139		−0.20 (−0.51–0.11) 0.208		−0.30 (−1.01–0.48) 0.474	
0.2 m/s	0.07 (−0.19–0.33) 0.575		−0.15 (−0.34–0.03) 0.107		0.63 (−1.41–2.67) 0.537		0.49 (−0.19–1.18) 0.151		0.33 (−0.03–0.70) 0.071		0.63 (−1.36–2.62) 0.523	
Long tests												
Duration (hours:minutes)	14:28		14:24		NA		15:13		15:18		4:46	
Number of measurements per experiment (N)	5256		5316				5446		5658		1752	
40 °C, 70% humidity	Eco-cooler effect estimates for temperature ( °C)											
4.0 m/s	0.02 (−0.05–0.09) 0.562		0.11 (0.08–0.14) <0.001		NA		0.10 (0.07–0.13) <0.001		0.09 (0.06–0.12) <0.001		0.09 (0.01–0.18) 0.028	
40 °C, 70% humidity	Eco-cooler effect estimates for humidity (%)											
4.0 m/s	−0.28 (−0.53 to −0.02) 0.032		−0.44 (−0.67 to −0.20) <0.001		NA		−0.33 (−0.77–0.11) 0.146		−0.29 (−0.56 to −0.02) 0.033		−0.28 (−0.68–0.13) 0.179	
Wind speed	Wind speed after eco-cooler (m/s)											
	Design 1		Design 2		Design 3		Design 4		Design 5		Design 6	
	A	B	A	B	A	B	A	B	A	B	A	B
4.0 m/s	1.41	0.69	3.80	**4.01**	1.29	1.34	2.75	3.30	1.85	2.03	3.14	3.23
2.0 m/s	1.02	0.50	**2.02**	**2.02**	0.65	0.66	1.65	1.77	0.96	1.06	1.58	1.65
0.2 m/s	0.00	0.00	0.00	0.00	0.00	0.00	0.00	0.00	0.00	0.00	0.00	0.00

The effects of the eco-cooler on temperature and humidity were measured with six sensors placed in the climate chambers. Three of these sensors were placed before the eco-cooler and three after the eco-cooler. We used linear regression to quantify the eco-cooler effect (i.e., the difference between the sensor measurements before and after the eco-cooler). The numbers shown for each eco-cooler design and climate scenario are (from right to left in each cell): the eco-cooler average effect size, and the 95% confidence interval (in parentheses) and the p value of the effect size.

Wind speed and atmospheric pressure estimates were empirical spot measurements.

High humidity = 70%, medium humidity = 60%, low humidity = 40%.

High temperature = 40 °C, medium temperature = 35 °C, low temperature = 30 °C.

Difference in means are significant if temperature is ±0.5 °C and humidity is ±4.5%.

Short duration tests consisted of three wind speeds: 4 m/s, 2 m/s and 0.2 m/s. Long duration tests consisted of one wind speed: 4 m/s.

Wind speed tests corresponding to A and B were conducted under climatic chamber conditions of 70% humidity, 40 °C, and 60% humidity, 35 °C respectively. Bolded numbers represent an increase in wind speed after the eco-cooler.

### Eco-cooler wind speeds in the open environment

The presence of the eco-cooler prevented air flow, reducing the wind chill effect. At the highest wind speed, less than 77% of the air flowed through the eco-cooler. Only one eco-cooler design (2), featuring a greater quantity of small bottles embedded into the board, showed higher wind speed following passage through the eco-cooler. Design 2, with incoming conditions of 4.0 m s^−1^ wind speed, 60% humidity, 35 °C, was able to maintain wind speed at 4.01 m s^−1^. Incoming wind speed of 2.0 m s^−1^, with both temperature and humidity variations, increased to 2.02 m s^−1^ following passage through the eco-cooler. Design 2 featuring more bottles and holes in the board was best at maintaining incoming wind speed, whereas most other designs impeded the airflow. Design 4 (doubled nozzle length) and design 6 (holes on the curved area of the bottles) let in between 69% to 89% of the airflow at 2.0 m s^−1^ and 4.0 m s^−1^, respectively.

Our open-environment study (Table 3) also demonstrated that wind speeds diminished as the air passed through the eco-cooler. Air speed was maintained at close proximity (up to 4.6 cm) to the centre line of the nozzle but decreased rapidly with distance (halved by 23.0 cm from the nozzle). Midway between the holes, we detected lower wind speeds close to the eco-cooler and the difference diminished with distance relative to the centre of the nozzle.

**Table 3:** Wind speeds at different wind intensities and distances from the eco-cooler measured in the open environment

Measurement position	Velocity (m/s)	
	From centre of nozzle	Midway between nozzle
*2.1* *m/s wind speed*		
Distance (cm)		
2.3	2.00	-
4.6	1.90	-
9.2	1.60	0.40
23.0	1.30	0.70
24.0	0.70	0.80
*4.1* *m/s wind speed*		
Distance (cm)		
2.3	4.70	-
4.6	4.60	-
9.2	3.90	0.80
23.0	2.00	1.60
24.0	1.80	1.50
*10.6* *m/s wind speed*		
Distance (cm)		
2.3	10.1	-
4.6	10.4	-
9.2	8.90	4.10
23.0	4.70	3.80
24.0	4.50	3.70

Error range of air velocity is ±0.3 m/s.

The 24.0 cm positions are measured by a hotwire device.

Ambient air conditions: room temperature (18.7 °C) and atmospheric pressure of  102.1 kPa.

**Fig. 1:**
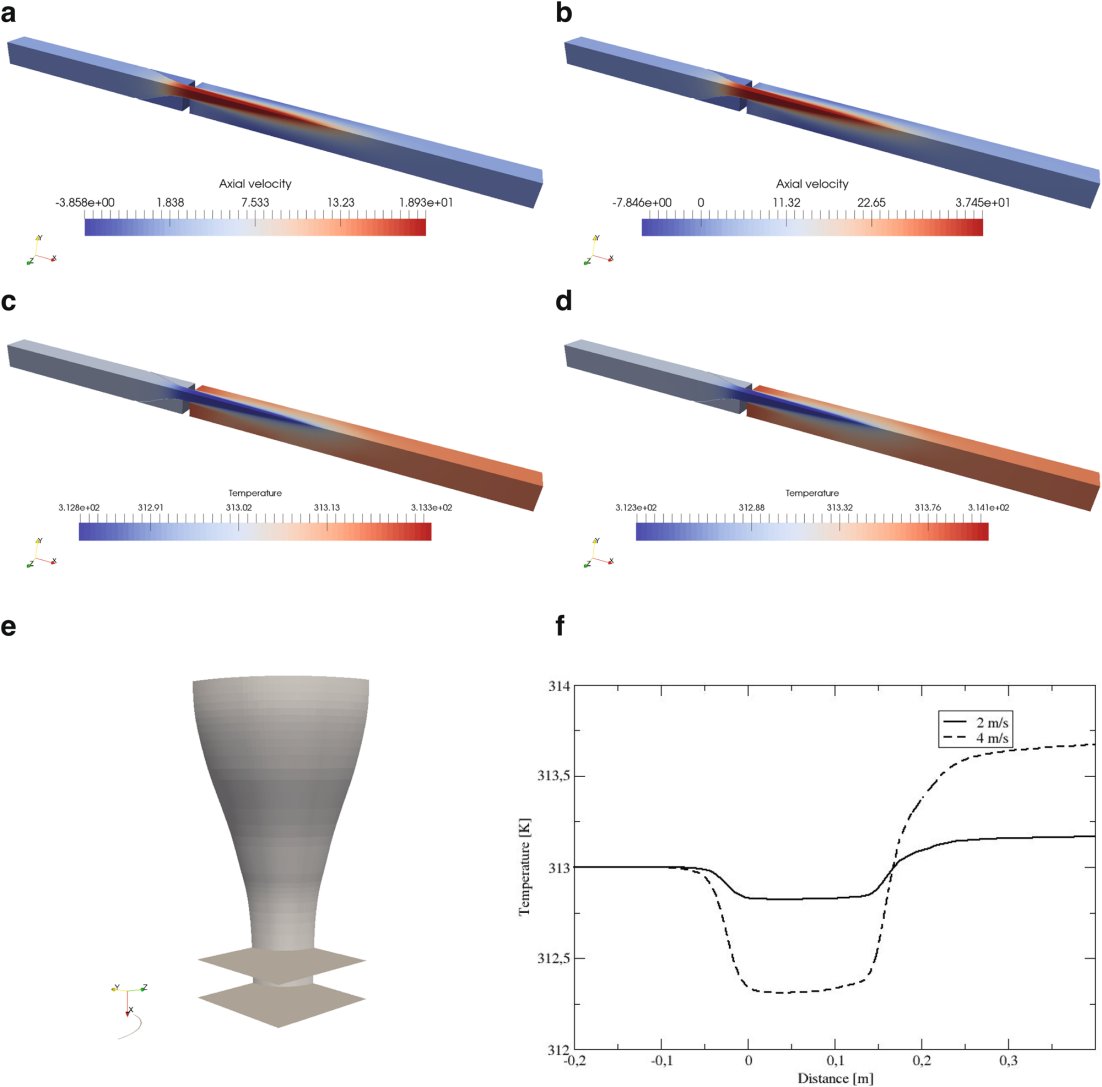
Simulation of airflow velocity and temperature at the nozzle in the wind tunnel. Panels **a**, **b** are the axial velocity at 2.0 m s^−1^and 4.0 m s^−1^, respectively. Panels **c**, **d** are the temperature distribution through the nozzle at 2.0 m s^−1^ and 4.0 m s^−1^, respectively. Panel **e** depicts the cross-section of the nozzle used in the simulation. Panel **f** presents the temperature change (*y* axis) with distance (*x* axis) along the nozzle centre line.

In our accompanying theoretical modeling study, higher air velocity entering the eco-cooler resulted in greater exit air velocity and temperature differential (cooling). Assuming a static air inflow temperature of 313 K (40 °C), we calculated the maximum cooling of 0.35 °C at 2.0 m s^−1^ incoming air velocity (Fig. 1a, and c) and 0.85 °C at 4.0 m s^−1^ incoming air velocity (Fig. 1b, d). Our calculations, which used the empirical wind tunnel data, revealed that the reduction in temperature occurred in close proximity to the nozzle due to *vena contracta*^25^, i.e., the smallest cross-section of the air stream is where the velocity of the flow is greatest. We show a cross section of the nozzle assumed in our calculations in Fig. 1e. Spreading of the air jet from the centre line away from the nozzle caused velocity to decrease in the flow direction and temperature to rise again (Fig. 1f).

We calculated that the exit pressure, relative to entry pressure needed to achieve a temperature decrease of 5 °C through the eco-cooler is 95.8 kPa. The 95.8 kPa pressure difference is equivalent to requiring an entry air velocity into the eco-cooler of 0 m s^−1^ and an exit velocity from the nozzle of 100 m s^−1^. To contrast, a stronger entry wind velocity (30 m s^−1^, almost the speed of tropical cyclones) requires exit velocity of 105 m s^−1^, assuming the following: atmospheric pressure *p*_atm_ = 101.3 kPa, ambient temperature *T*_atm_ = 313 K (40 °C), specific heat capacity *c*_*p*_ = 1004.5 J kg^−1^ K^−1^ and isentropic exponent *k* = 1.4 for air. To put this into perspective, *p*_exit_ = 95.8 kPa is equivalent to the atmospheric pressure at an elevation of 500 m above mean sea level^19^, which is on par with the height of the One World Trade Center, New York City (541 m high). These values do not match the climate conditions in Bangladesh where wind speeds generally do not exceed 4.0 m s^−1^.

## Discussion

We conducted the first test of the eco-cooler in a wind tunnel and an open-environment laboratory study. Unlike previous studies of the eco-cooler, which used less reliable methods^18–22^, our results demonstrated that this frugal innovation does not significantly increase wind speed or reduce temperature. The eco-cooler is thus unlikely to be effective as a passive home cooling device. The negligible cooling effect we observed in some of our trials did not extend beyond the nozzle of the eco-cooler. Using a theoretical model and our empirical data, we also showed that a constant atmospheric pressure difference of 6000 Pa between the inside and outside of a house—which is extremely unlikely to occur naturally—is required for a 5 °C temperature reduction. This pressure difference is equivalent to maintaining a wind speed of 0 m s^−1^entering the eco-cooler and 100 m s^−1^ exiting the eco-cooler. The premise of the eco-cooler is that the passage of air through narrow bottle nozzles creates a high-pressure gradient, increases the velocity of air flow, and lowers air temperature. We did not observe such effects beyond the nozzles.

Furthermore, we found that altering the bottle designs and increasing the time of eco-cooler use did not influence the cooling performance. Our results differed from those of previous studies^18–22^ because we empirically mimicked the real-life weather conditions of Bangladesh in a climatic chamber. According to the Bangladesh Meteorological Department (2022), monthly average wind speeds across five villages reported to have installed the eco-cooler ranged between 0.82 m s^−1^ (Daulatdia) and 3.74 m s^−1^ (Paturia), while the wind speeds we used in our wind tunnel experiments ranged from 0.2 m s^−1^ to 4.0 m s^−1^. Wind speeds recorded at weather stations from these villages were close to the wind speed of 2.0 m s^−1^, which we used in our study. If the eco-cooler were to induce temperature changes this is more likely to be at higher wind speeds; we thus included the wind speed of 4.0 m s^−^^1^ in our experiments. Finally, unlike previous studies^18,20,22^, which modified the eco-cooler design and used an enhancer to achieve a cooling effect, we tested the original design of the eco-cooler, which is currently used in real life application in Bangladesh. The modifications to the original design substantially increase both the cost and complexity of the eco-cooler, limiting its usefulness for real-life application in resource-poor communities^22^.

Electric fans are a cheaper alternative to air conditioners, and are owned by 86% of urban and 49% of rural households in Bangladesh^26^. Electric fans create a wind chill effect to enhance evaporative cooling^27^ and produce quantifiable health benefits such as delaying the onset of cardiovascular strain during heat stress under certain temperature thresholds^28^. However, electric fans require access to electricity and many resource-poor communities (e.g., people living in informal settlements) have limited access to electricity^29^. Therefore, it is vital to invest resources in the development of passive cooling innovations that can reduce indoor ambient air temperature. Recently, some of the resource-richest parts of the world (e.g., the West Coast of USA and Canada) have experienced extreme heat waves exacerbated by anthropogenic climate change^30^, which will likely boost the global demand for effective passive cooling solutions. Across a wide spectrum of communities worldwide, climate change and associated heat waves have a detrimental effect on public health, comfort and productivity^31^^,32^.

A major strength of our study is that we conducted three comprehensive scientific studies to establish the effectiveness of the eco-cooler as a passive home cooler: i) wind tunnel experiments, ii) open-environment laboratory study, and iii) a theoretical model. The empirical experiments enabled us to mimic the climate conditions in Bangladesh and test how varying these conditions affected eco-cooler performance. We also investigated the effects of varying eco-cooler designs. We designed the open-environment laboratory study to measure the distances to which the eco-cooler changed wind speed from the bottle nozzles into a room. Finally, in our theoretical modelling study we aimed to determine the conditions required to observe a 5 °C temperature reduction with eco-cooler use. We demonstrated that the original eco-cooler design is unlikely to create a substantial temperature reduction. Our empirical findings show that the greatest reduction of temperature was ~0.20 °C, which occurred at the lowest incoming wind speed of 0.2 m s^−1^—an almost negligible effect. Several scenarios resulted in temperature increases of a similar size (up to 0.2 °C). In the open-environment laboratory study, a 0.85 °C temperature reduction at 4.0 m s^−1^ incoming wind speed did not last beyond the nozzle of the eco-cooler. Our theoretical model demonstrated that an exit pressure of 95.8 kPa, which is equivalent to an exit air velocity of 100 m s^−1^ (relative to entry wind velocity of 1 m s^−1^) is required to cool the indoor temperature by 5 °C with eco-cooler use. These conditions rarely occur in terrestrial zones inhabited by humans.

Our research has several limitations. Our tests were not conducted on eco-coolers deployed in the field in Bangladesh—however, we used publicly available blueprints to construct new eco-coolers that closely resemble the ones deployed in real life. Because our empirical research took place in laboratory environments, we may not have captured some environmental or built environment factors that can affect the cooling performance of the eco-cooler in real life. Our empirical trial in the wind tunnel and climate chamber, however, did mimic Bangladeshi weather. Finally, we only tested the eco-cooler without modifications. Future studies could evaluate the eco-cooler with modifications, in particular those that are likely feasible and affordable in resource-poor communities^18,20,22^.

In our current study, we have focused on the absolute temperature of the air (which is a measure of thermal energy content) and its coupling to the kinetic energy of the air motion. Without any external influences (such as mechanical work or heat transfer to the surroundings) and neglecting any internal friction in the flow, these two energy types are in balance, resulting in an increase in kinetic energy and a corresponding decrease in thermal energy (and temperature) and vice versa. This is the likely mode of operation of the eco-cooler. Further lowering of temperature is plausible, however, if more thermal energy can be extracted from the air—for example through evaporation of water. Indeed, this is the mechanism used in many commercial electric air coolers for domestic use. For evaporative cooling, however, extremely dry air is necessary to maximise evaporation; we do not see how evaporation can be used in conjunction with the eco-cooler to achieve passive cooling, and therefore did not investigate this. Increasing the wind chill can also enhance cooling. Here, apparent temperature or heat index, which accounts for humidity, wind speed and thermal radiation can also be used to gauge the temperature perceived by people and is mainly used in outdoor conditions to calculate the so-called wind chill effect, i.e., the decrease in perceived air temperature when wind carries heat away from the human body. However, since the eco-cooler, when fitted in a window frame or similar, would not accelerate the airflow into a dwelling (but rather act as a flow restricter), an open window frame would probably generate a higher wind chill effect since it would let the wind pass through almost unhindered. Further research can trial passive cooling technologies that are feasible in resource-poor communities, such as the cool roofs^33^ and strategic shading of buildings^34^ to quantify their effects on indoor climate and human wellbeing and health^34^.

Based on our results, there is no indication that the eco-cooler is an effective passive home cooler. The public response to the eco-cooler is encouraging for the future of locally designed, sustainable innovations to some of the world’s most pressing problems, such as climate change. Entrepreneurs should continue to identify, produce, and promote evidence based innovations. It is also important that passive cooling technologies and other frugal innovations are more rigorously tested prior to deployment in real life.

## Methods

### Wind tunnel experiments

**Fig. 2:**
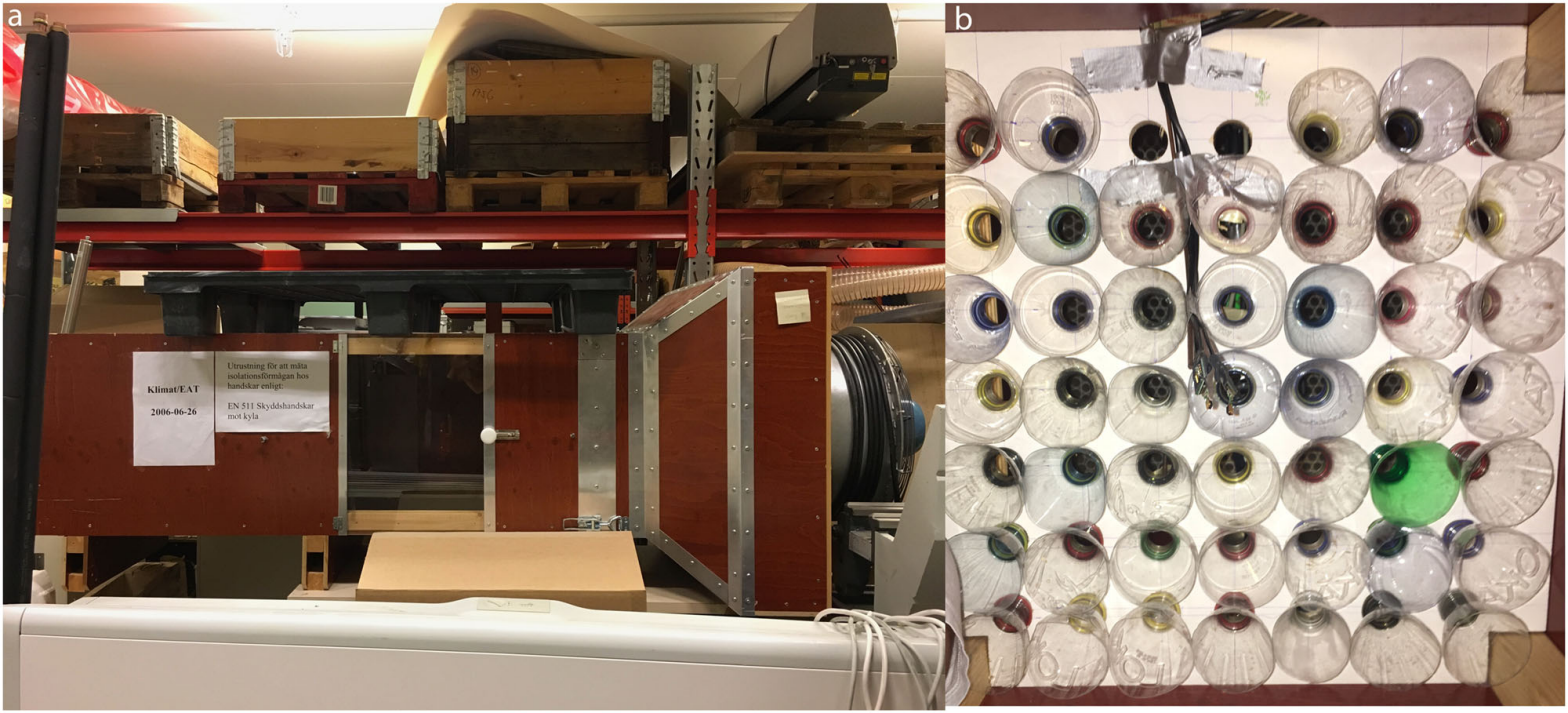
The wind tunnel set-up used for the experiments. Panel **a** depicts the full wind tunnel, panel **b** depicts the eco-cooler set up including sensors inside the wind tunnel.

For our experiments, we set up the wind tunnel inside a climatic chamber at Lund University, Sweden, and simulated Bangladeshi climate conditions (Fig. 2). We built three eco-cooler boards, and tested six different bottle designs (Supplementary Table 2 and Supplementary Figs. 1–6). The preliminary tests were designed to establish how long it would take for the chamber temperature and humidity to stabilise after the climate parameters were altered, and whether the hypothesised cooling through the eco-cooler was immediate or delayed.

Climate conditions in the chamber were based on real weather data from 2016 obtained from the National Climatic Data Centre in Bangladesh. We conducted 92 experiments in total (*N* = 7686 measurements in 87 short experiments and *N* = 23,428 measurements in 5 long experiments). The respective high, medium and low measures were for temperature (40 °C, 35 °C, 30 °C), humidity (70%, 60%, 40%), and wind speed (4.0 m s^−1^, 2.0 m s^−1^, 0.2 m s^−1^). We conducted experiments in two stages. In the first set of experiments, we tested 27 climate combinations with high, medium and low intensities of temperature, humidity and wind speed for 20 minutes each to identify if the eco-cooler performed better in certain climate conditions (27 experiments with 126 measurements each). In the second set of experiments, we tested the efficacy of the eco-cooler with six different bottle designs under two climate conditions (40 °C, 70% humidity and 35 °C, 60% humidity) for 20 minute bursts (60 experiments with 126 measurements each). In addition, we conducted overnight experiments at 40 °C temperature, 70% humidity and 2.0 m s^−1^ wind speed for five different bottle designs to test the efficacy over long durations (five experiments with 5,256, 5,316, 5,446, 5,658, 1,752 measurements, respectively).

**Fig. 3:**
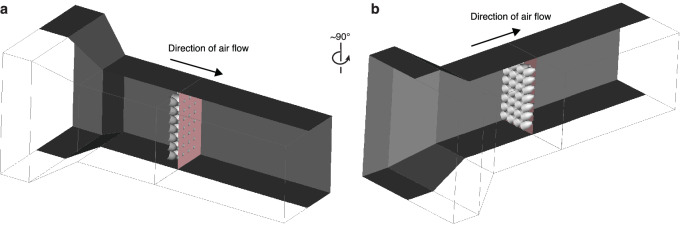
3D representations of the eco-cooler and its position in the wind tunnel. Panel **a** indicates exit direction of air flow and panel **b** indicates the entry direction of air flow.

We placed the eco-cooler (50 cm × 50 cm) in the middle of an open circuit wind tunnel (Fig. 3 and Supplementary Fig. 7) inside a climatic chamber. The fan simulated wind blowing into the inlet chamber, through the eco-cooler, and exiting the outlet chamber at set velocities; 4.0 m s^−1^ (21 Hz), 2.0 m s^−1^ (11 Hz) and 0.2 m s^−1^ (1 Hz). In addition to wind blowing into the chamber, the fan can simulate air suction to create negative pressure in the chamber. We placed two sets of temperature and humidity sensors (Sensirion AG, Switzerland; accuracy ±0.5 °C, ±4.5% relative humidity) in the inlet and outlet of the wind tunnel and one set of sensors in the surrounding chamber. Each sensor set consisted of three spot measurements at the inlet, which were located: (i) prior to the nozzles of the eco-cooler at the outlet, (ii) 24 cm from the eco-cooler, and (iii) in the middle of the climatic chamber away from the wind tunnel. The third location served as a control for the surrounding climate. We spot-measured pressure using a metre from GE Druck Standards Laboratory, Leicester, UK, by inserting the probe into the wind tunnel through a small access hole. We measured the airflow 24 cm after the eco-cooler with an Air Flow^TM^ metre (Developments Ltd AV-2, High Wycombe, England, UK) to determine whether the eco-cooler hindered or accelerated the air flow. The tubes at the end of the outlet created homogeneous airflow through the wind tunnel.

For the statistical analysis, we averaged measurements from three temperature and humidity sensors positioned before and after the eco-cooler at one-minute intervals. We estimated the mean change in the coefficients for temperature or humidity after the eco-cooler, relative to before the eco-cooler, using linear regression. Effect estimates with *p* values below 0.05 were deemed statistically significant. The temperature and humidity sensors had an error margin of ±0.5 °C and ±4.5% respectively. We performed statistical analyses using *R* v. 3.3.3.

To simulate nozzle flow in the wind tunnel, we employed computational fluid dynamics to better understand the flow downstream of the nozzles in the wind tunnel (details presented in Supplementary Figs. 8 and 9).

### Open-environment laboratory study

**Fig. 4:**
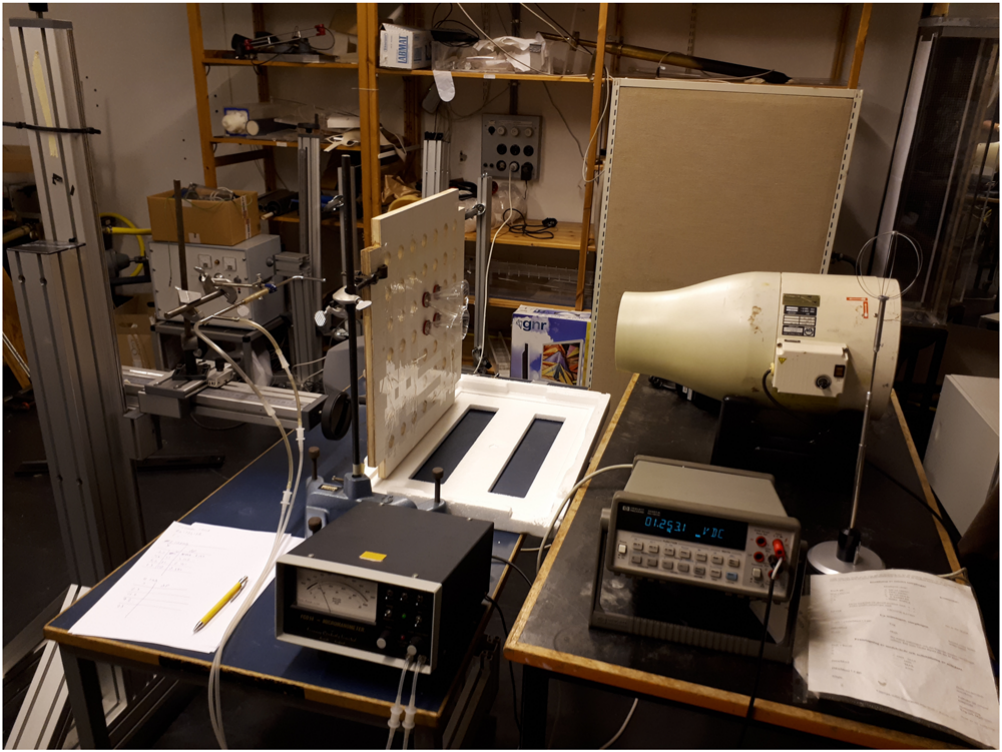
The open environment test set-up. The air flow in the direction of the eco-cooler is controlled in the experimental set-up.

**Fig. 5:**
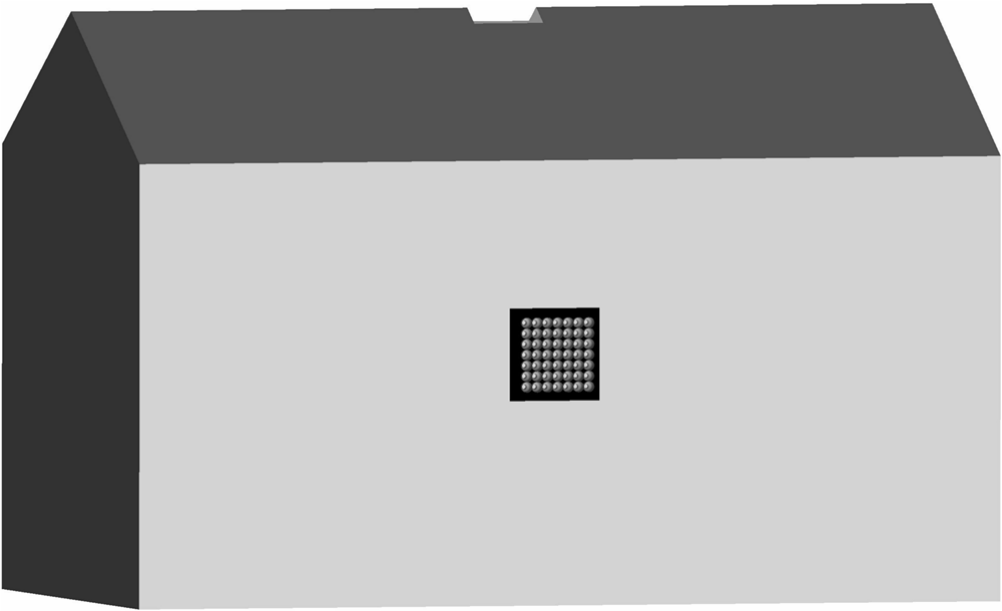
A computer-aided image of the eco-cooler fitted on a window frame. The eco-cooler is positioned at the centre of the wall in this example.

To complement the closed wind tunnel studies where airflow and acceleration through the eco-cooler are governed by mass conservation, we also performed experiments in an open environment at room temperature (18.7 °C) and at 102.1 kPa. Airflow of 10.6 m s^−1^, 4.1 m s^−1^ and 2.1 m s^−1^ were generated by an open wind tunnel (Leybold Didactic GmbH, Germany) 30 cm upstream from the eco-cooler. To match the diameter of the open wind tunnel (0.15 m), we fit four bottles on the eco-cooler board (Fig. 4 and Supplementary Fig. 10), which ensured that a uniform flow entered the bottles. Using four bottles did not alter the flow through each bottle at the proximal part of the jet, compared to the full set-up. Far downstream where the jets interacted, however, the flow situation varied. Wind speed was measured along the centre of the flow path at points 2.3 cm, 4.6 cm, and 9.2 cm downstream of the eco-cooler using a Pitot static tube connected to a FCO14 micromanometer (Furness Controls Ltd, UK: accuracy ±0.5%). The signal from the manometer (0–5 V) was sampled by a multimeter (34401 A, Hewlett Packard, USA), which was used to calculate an average of 200 samples for each measurement point. We also measured wind speed midway between the two holes at 9.2 cm and 23 cm. Additionally, the Air Flow^™^ metre and hotwire probe (Semwa Air 300, Semwa AB) were used to measure the flow speed 24 cm downstream of the eco-cooler as well as at the centre and midway between the nozzles. We illustrate how an eco-cooler, in a real building application, can be fitted to a window frame or any other opening on an outer wall (Fig. 5).

### Theoretical model of a home setting

If the eco-cooler exit temperature is lower than the ambient temperature, the eco-cooler exit pressure will have to be lower than the atmospheric pressure. We calculated the exit speed required for a certain exit temperature through the adiabatic energy equation (detailed theoretical calculations explaining Equations (1–5) are outlined in the Supplementary file (Theoretical calculations in a home setting). Reformulating Equation (3) in the Supplementary file, the exit speed is calculated for a given exit temperature and wind speed (Eq. (6)).

6 $${V}_{{\rm{exit}}}=\sqrt{{2c}_{p}\left({T}_{{\rm{atm}}}-{T}_{{\rm{exit}}}\right)+{V}_{\rm{wind}}^{2}}$$

We calculated the pressure inside the building required to achieve a certain temperature difference using Eq. (7). Here, we use the constancy of the stagnation properties (*p*_0_, *T*_0_) as shown in Equation (4), in the Supplementary materials. Hence, without a difference in pressure, *T*_exit_ will equal *T*_atm_ and *V*_exit_ will equal *V*_wind_.

7 $${p}_{{\rm{exit}}}={p}_{{\rm{atm}}}{\left(\frac{{T}_{{\rm{exit}}}}{{T}_{{\rm{atm}}}}\right)}^{\frac{k}{k-1}}$$

Also note that even though *p*_0_ and *T*_0_ will vary with the wind speed, the exit pressure will be the same independent of wind speed. We also emphasise that the exit pressure required for any wind speed would be even lower if losses in mechanical energy are also considered.

## Supplementary information

Supplementary Information

## Data Availability

Data used for the replication of our analysis is publicly available at: https://github.com/PrasadLiyanage/Eco-Cooler.git.

## Code availability

Code used for the replication of our analysis is publicly available at: https://github.com/PrasadLiyanage/Eco-Cooler.git.

## References

[1] Kuo, A. Harnessing frugal innovation to foster clean technologies. *Clean. Technol. Environ. Policy* **19**, 1109–1120 (2017).

[2] Prabhu, J. Frugal innovation: doing more with less for more. *Philos. Trans. A Math. Phys. Eng. Sci.* **375**, 2095 (2017). 10.1098/rsta.2016.0372 28461436

[3] Fredriksson, E. & Tömmervik, J. *Frugal is the new innovative thinking: a qualitative study of frugal innovations and sustainable development in resource-poor environments*. (Mälardalen University, Sweden, 2014).

[4] Sissoko, M. & Castiaux, A. *How does frugal innovation emerge and lead to sustainability in developing countries? A case study in Malian agricultural areas* in *European Association of Agricultural Economists*. Galway, West of Ireland. 10.22004/ag.econ.276195 (2018).

[5] Weyrauch, T. & Herstatt, C. What is frugal innovation? Three defining criteria. *J. Frugal Innov.* **2**, 1 (2016).

[6] Schumacher, E. F. *Small is beautiful*. (Random House, 2011).

[7] Joshi, S. This ingenious no-electricity cooler made with plastic bottles is helping Bangladesh cope with summer. *Mashable* https://mashable.com/article/eco-cooler-plastic-bottles-no-electricity (2016).

[8] GreyAdvertising-Bangladesh, *Eco-Cooler | Grey Dhaka unveils world’s first zero-electricity air cooler made from plastic bottles*. (2017).

[9] GreyGroup. *Eco Cooler*. Available from: http://grey.com/apac/work/key/eco-cooler/id/12475/ (2017).

[10] Nowshin, N. Zero-power cooling, with plastic. *The Hindu* https://www.thehindu.com/specials/impact-journalism-day-2017/eco-cooler-the-worlds-first-ever-zero-electricity-air-conditioner-made-out-of-plastic-bottles/article19108375.ece (2017).

[11] Owano, N. Household cooling device is designed to run without electricity. *TechnXplore* https://techxplore.com/news/2016-06-household-cooling-device-electricity.html (2016).

[12] Forum, W. E. A Breath of fresh air. *Facebook* https://www.facebook.com/worldeconomicforum/videos/10154773053621479/ (17 Sept 2017).

[13] Islam, N. Sustainability issues in urban housing in a low-income country: Bangladesh. *Habitat Int.* **20**, 377–388 (1996).

[14] Nahiduzzaman, K. M. & Haas, T. Micro climatic house design: a way to adapt to climate change? the case of Ghar Kumarpur village in Bangladesh. *Theor. Empir. Res. Urban Manag.* **3**, 9 (2008).

[15] Hondula, D. M., Rocklöv, J. & Sankoh, O. Past, present, and future climate at select INDEPTH member health and demographic surveillance systems in Africa and Asia. *Glob. Health Action*. **5**, 74–86 (2012). 10.3402/gha.v5i0.19083 PMC3508753 23195511

[16] Cooper, R. G. & Kleinschmidt, E. J. Determinants of timeliness in product development. *J. Prod. Innov. Manag.* **11**, 381–396 (1994).

[17] Schmidt, C. W. & Grossmann, I. E. Optimization models for the scheduling of testing tasks in new product development. *Ind. Eng. Chem. Res.* **35**, 3498–3510 (1996).

[18] Ch, B. & Mummina, V. Performance evaluation of an eco-cooler analysed by varying the physical and flow parameters. *IOP Conf. Ser. Mater. Sci. Eng.* **377**, 012024 (2018).

[19] Khan, A. H. et al. Evaluation of cooling capability of an eco-cooler: experimental and numerical analyses. *Energy Procedia* **160**, 100–107 (2019).

[20] Mishra, A., Gupta, A. D., Mandal, A. K. & Singh, A. Zero electricity air conditioning using phase changing materials. *Int. J. Therm. Eng.* **4**, 1 (2016).

[21] Sathyakumar, N., Aswath, S., Prakash, R. K. & Bharathi, R. M. Smart technology to reduce internal room temperature. *Int. Res. J. Eng. Tech.* **6**, 3 (2019).

[22] Karunakarareddy, L. & Hemachandra Reddy, K. Production of cost effective cooling using eco-cooler. *Int. J. Tech. Innov. Mod. Eng. Sci.* **3**, 11 (2017).

[23] Li, M., Peterson, H. B. & Coimbra, C. F. Radiative cooling resource maps for the contiguous United States. *J. Renew. Sustain. Energy* **11**, 3 (2019).

[24] Otto, K. N. *Product design: techniques in reverse engineering and new product development* (Tsinghua University Press, 2003).

[25] Massey, B. S. & Ward-Smith, A. J. *Mechanics of Fluids*. 9th edn. (Spoon Press, 2012).

[26] National Institute of Population Research and Training (NIPORT). *Bangladesh Demographic and Health Survey 2014*. (NIPORT, Mitra Associates and ICF International, 2016).

[27] Department of Energy. *Cooling Your Home with Fans and Ventilation*. (National Renewable Energy Laboratory, 2001).

[28] Jay, O. et al. Should electric fans be used during a heat wave? *Appl. Ergon.* **46**, 137–143 (2015). 25134988 10.1016/j.apergo.2014.07.013

[29] Patel, N. Figure of the week: electricity access in Africa. *The Brookings Institution*. https://www.brookings.edu/articles/figure-of-the-week-electricity-access-in-africa/ (2019).

[30] The Economist. The danger posed by heatwaves deserves to be taken more seriously. *The Economist*. https://www.economist.com/leaders/2021/07/03/the-danger-posed-by-heatwaves-deserves-to-be-taken-more-seriously (July 2021).

[31] Bastos, A. et al. Direct and seasonal legacy effects of the 2018 heat wave and drought on European ecosystem productivity. *Sci. Adv.* **6**, 24 (2020). 10.1126/sciadv.aba2724 PMC7286671 32577519

[32] Royé, D. et al. Heat wave intensity and daily mortality in four of the largest cities of Spain. *Environ. Res.* **182**, 109027 (2020). 31884190 10.1016/j.envres.2019.109027

[33] Kolokotroni, M. et al. Cool roofs: high tech low cost solution for energy efficiency and thermal comfort in low rise low income houses in high solar radiation countries. *Energy Build* **176**, 58–70 (2018).

[34] Santamouris, M. et al. Recent progress on passive cooling techniques: advanced technological developments to improve survivability levels in low-income households. *Energy Build* **39**, 859–866 (2007).

