# Supplementary material for: Evaluating the effectiveness of the ‘eco-cooler’ for passive home cooling
Source: NPJ Clim Action. 2024 Nov 2;3(1):94. doi: 10.1038/s44168-024-00165-7 (PMC11531403; doi:10.1038/s44168-024-00165-7)
Supplement: Supplementary file 1 — Supplementary Information [file 44168_2024_165_MOESM1_ESM.pdf]

Supplementary File

Evaluating the Effectiveness of the 'eco-cooler' for passive home cooling

Aditi Bunker, Karin Lundgrun Kownacki, Sudipa Sarker, Rahmatul Bari, Malabika Sarker, Jonathan J. Buonocore, Pascal Geldsetzer, Johan Revstedt, Till Bärnighausen

Supplementary Table 1 Wind speeds (m/s) in regions of eco-cooler deployment

Town	Station	Jan	Feb	Mar	Apr	May	Jun	Jul	Aug	Sep	Oct	Nov	Dec	Annual
Daulatdia	Chuadanga	0.82	0.95	1.57	2.47	2.44	2.31	1.99	1.71	1.55	0.94	0.62	0.66	1.50
Nilphamari	Dinajpur	0.98	1.20	1.72	1.99	1.76	1.74	1.62	1.43	1.18	0.81	0.66	0.69	1.32
Paturia	Khulna	1.34	1.61	2.48	3.72	3.74	3.34	3.20	3.25	2.23	1.27	0.97	1.07	2.38
Modonhati	Rajshahi	1.64	1.77	2.26	3.40	3.73	3.55	3.24	2.90	2.45	1.53	1.45	1.66	2.46
Khaleya	Rangpur	1.25	1.59	2.50	3.40	3.01	2.93	2.77	2.60	2.14	1.63	1.46	1.25	2.22

Source: Bangladesh Meteorological Department (https://live.bmd.gov.bd/p/Normal-Wind-Speed)

Supplementary Table 2 Six different bottle designs were tested

Bottle description	Theory behind design
1. Big bottles – 9 vents	Increased cross-section of the bottle slightly reduced pressure losses. Because flow is governed by the pressure difference, fewer vents (from the large bottles) resulted in higher flow speed in the wind tunnel system.
2. Small bottles – 49 vents	Increased number of vents and nozzles increases the mass air flow
3. Flipping board simulating air suction with bottles used in test two	Tested whether air suction performed better than air being blown into the cooler
4. Extension of the nozzle (bottle within a bottle)	Increased pressure reduction
5. Rounded edges on bottles	Decreased losses in air flow resistance
6. Small vents on curved area of bottle	Direction of air flow was altered when air contacts a curved surface, but flow into cooler is maintained

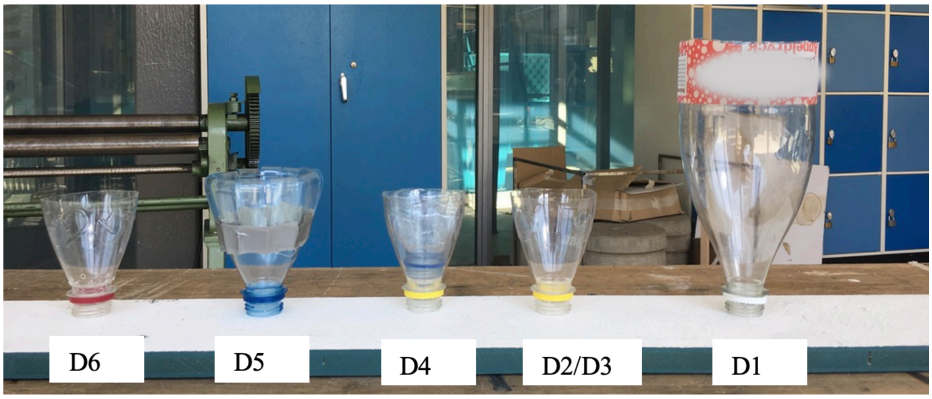

Supplementary Figure 1. Bottle design overview

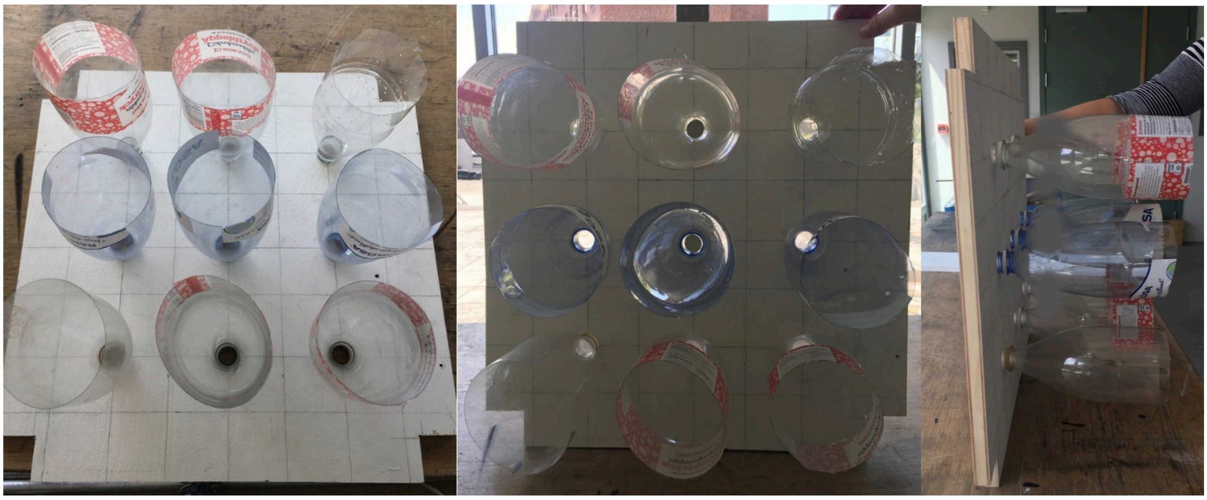

Supplementary Figure 2. Design 1: Large bottles

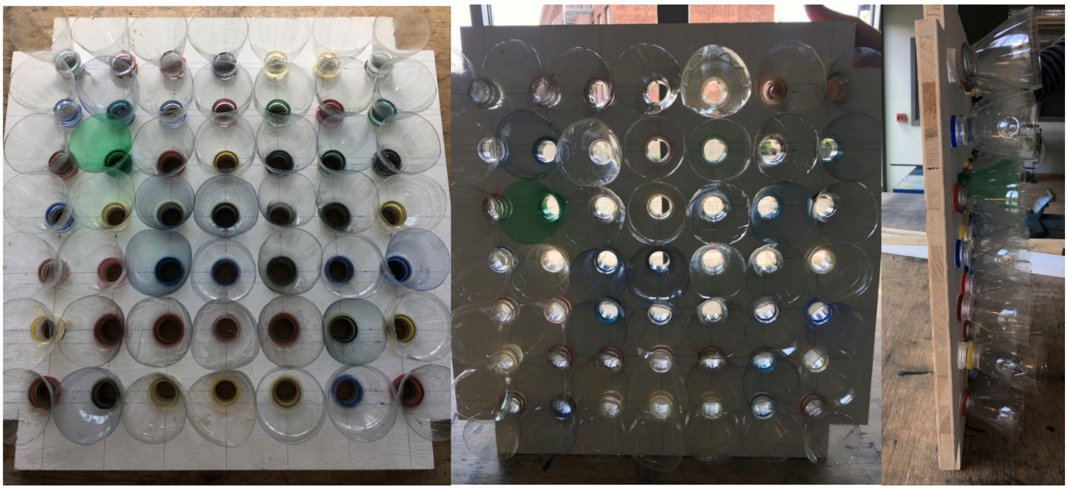

Supplementary Figure 3. Design 2/Design 3: Small bottles

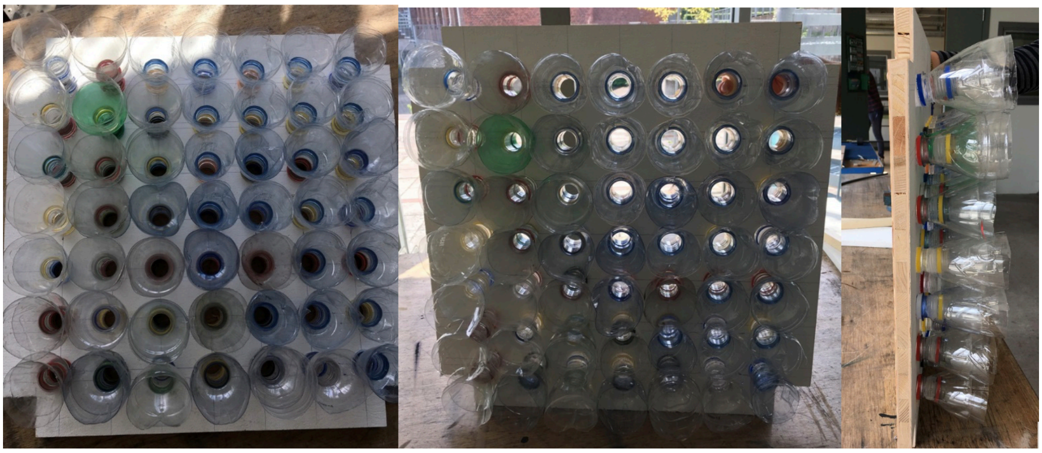

Supplementary Figure 4. Design 4: Extended nozzle length (bottle within bottle)

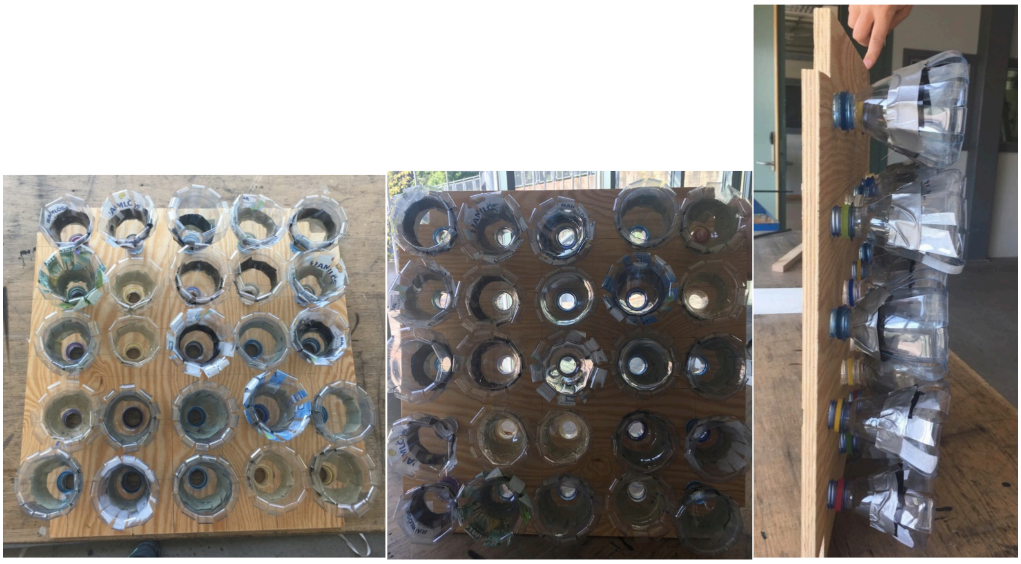

Supplementary Figure 5. Design 5: Reduced wind resistance (flower shape)

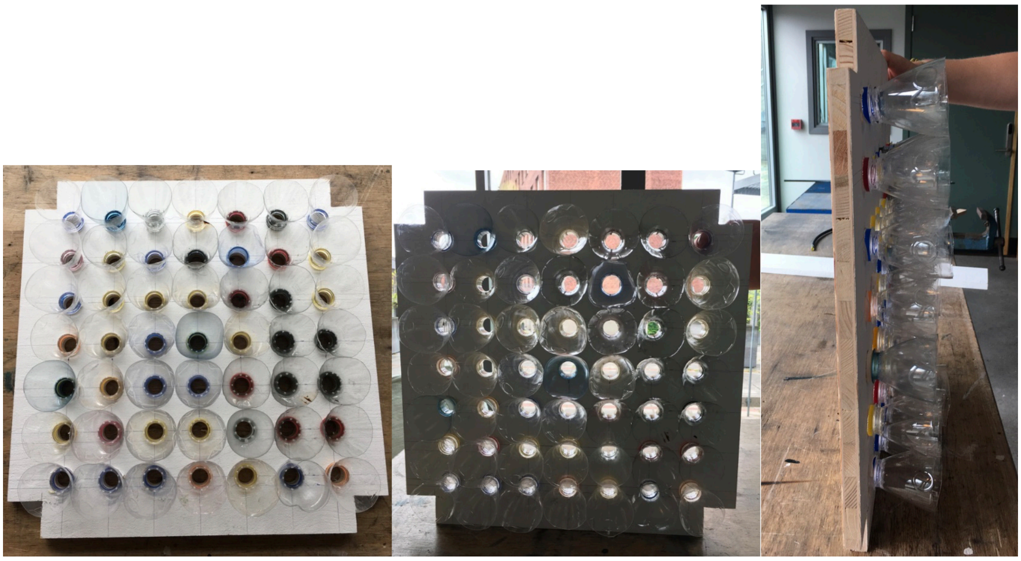

Supplementary Figure 6. Design 6: Holes in the nozzle to encourage mixing and increased air flow

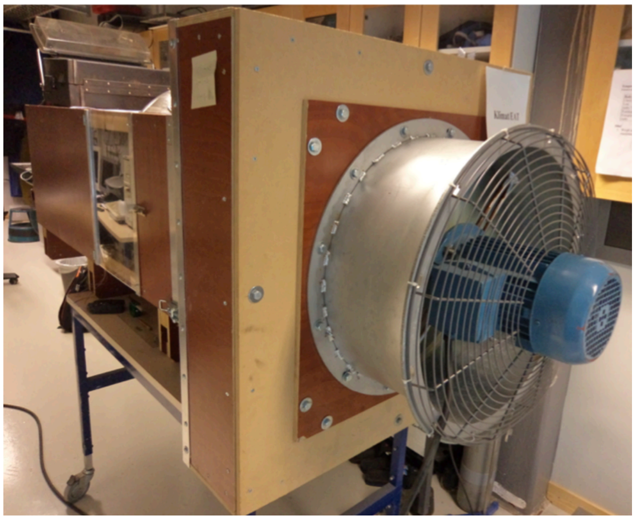

Supplementary Figure 7. Wind tunnel

Supplementary File S3: Details on methods for simulation of nozzle flow in the wind tunnel

The simulations are performed using the open-source software package OpenFOAM 4.1. The solver is rhoSimpleFoam and the k-ω SST turbulence model (Menter, 1994) is used. All equations are discretised using a second order upwind scheme. Two cases are considered, inflow velocity of 2.0 m/s and 4.0 m/s. In both cases the inflow static temperature is set to 313 K. At the outlet a zero-gradient condition is used for velocity and temperature and the pressure is set to 101 kPa. Only one nozzle is considered, and the presence of surrounding nozzles is emulated using symmetry boundary conditions. Likewise, since the flow is statistically stationary geometrical symmetries in the nozzle have been used and hence only a quarter nozzle is simulated, as is shown in Figure 1, Supplementary File S3. The mesh is generated using snappyHexMesh and is refined in the vicinity of the nozzle as is outlined in Figure 1, Supplementary File S3. The nozzle shape is shown in Figure 2, Supplementary File S3. It should be noted that the nozzle shape is not identical to the one used in Design 2 (small bottles), because it was drawn by free hand. However, the dimensions, in terms of inflow and outflow diameters and length, are the same as in Design 2 and the shape resembles a bottle neck.

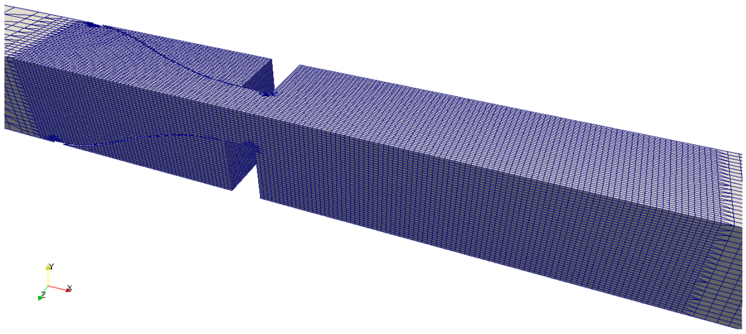

Supplementary Figure 8. Computational mesh in the region around the nozzle

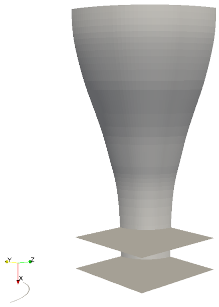

Supplementary Figure 9. Bottle (nozzle) geometry

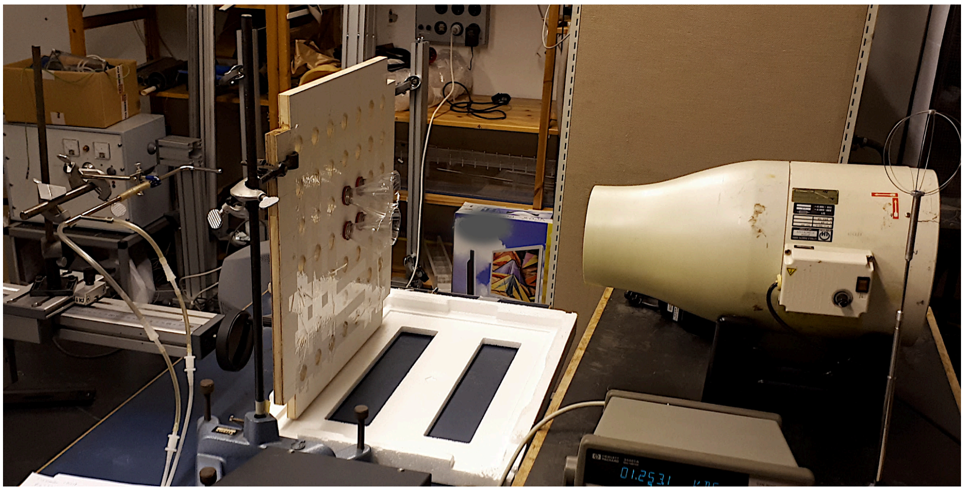

Supplementary Figure 10. Open environment test

Supplementary: Theoretical calculations in a home setting

Any fluid flow is governed by the conservation equation for mass, momentum and energy. The governing equations are usually simplified according to the flow situation, which is often characterised by non-dimensional numbers. One such number is the Mach number, which relates the flow speed to the speed of sound. For high Mach number flows through nozzles, it is common to assume one-dimensional isentropic flow. Hence, losses of energy are neglected and the velocity only varies in the flow direction. This allows for approximate, straight forward calculations of the flow through the nozzle. If the flow can be considered as isentropic (adiabatic and without losses), the relation between pressure and temperature is given by Equation 1 (1).

$$\frac{p_1}{p_2} = \left(\frac{T_1}{T_2}\right)^{\frac{k}{k-1}} \qquad \text{Equation (1)}$$

where 1 and 2 refer to different positions in the flow and k is the isentropic exponent.

In order to estimate the temperature change, the one-dimensional energy equation for adiabatic flow may be used (Equation 2)

$$c_p T_0 = c_p T_1 + \frac{V_1^2}{2} = c_p T_2 + \frac{V_2^2}{2} \qquad \text{Equation (2)}$$

where c_p is the heat capacity at constant pressure and T_0 is the stagnation temperature.

Using Equation 2 the outlet temperature of the flow can be calculated given known wind speed, V_{wind} (Equation 3).

$$T_{exit} = T_{atm} + \frac{V_{wind}^2}{2c_p} - \frac{V_{exit}^2}{2c_p} \qquad \text{Equation (3)}$$

where T_{atm} is the static temperature of the ambient air.

From Equation 3 one can conclude that to achieve an exit temperature lower than the surrounding static temperature the exit speed has to exceed the wind speed. Considering a situation where air flow is driven through the cooler in an open environment (e.g. the cooler mounted in a window frame) a lower static pressure is required on the inside of the building to achieve a lower temperature and, hence, a higher air speed at the exit point of the cooler relative to the entry. This can be seen by reformulating Equation 1 by using the stagnation values of pressure and temperature,

$$\frac{p}{p_0} = \left(\frac{T}{T_0}\right)^{\frac{k}{k-1}} \qquad \text{Equation (4)}$$

where p_0 and T_0 are constant throughout the flow in isentropic conditions. Since the stagnation conditions do not vary, Equation 4 can be used to formulate the conditions both outside the cooler and at the cooler exit. By combining these, one can get a direct connection between static pressure and temperature at the outside and at the cooler exit (Equation 5).

$$p_{exit} = p_0 \left(\frac{T_{exit}}{T_0}\right)^{\frac{k}{k-1}} = p_{atm} \left(\frac{T_0}{T_{atm}}\right)^{\frac{k}{k-1}} \left(\frac{T_{exit}}{T_0}\right)^{\frac{k}{k-1}} = p_{atm} \left(\frac{T_{exit}}{T_{atm}}\right)^{\frac{k}{k-1}} \qquad \text{Equation (5)}$$

In conclusion, if there is no pressure difference the exit temperature will be equal to the outside temperature and the exit speed will be equal to the wind speed. It should be noted that these equations do not account for the losses of mechanical energy that will occur in the nozzle. Also considering losses would result in lower exit speed and higher temperature.

Normally, these relations are only used in high Mach number flow situations, since for low Mach number flows the temperature is usually assumed constant. However, these equations are still applicable although the flow considered is at a low Mach number. However, the assumption of one-dimensional flow may be somewhat less valid. Comparing calculations using Equation 3 with CFD simulations of the LTH wind tunnel, one finds that for a wind speed of 4 m/s Equation 3 gives T_{exit}=311.6 K and the simulation T_{exit}=312.3 K.

References

1. Anderson, J. D. (2003). *Modern Compressible Flow with Historical Perspective*. McGraw Hill.
2. Menter, F. R. (1994). Two-equation eddy-viscosity turbulence models for engineering applications. *AIAA Journal*, *32*(8), 1598–1605. https://dio.org/10.2514/3.12149
